# A systematic review on time trend incidence of rheumatoid arthritis in outpatient rheumatology clinics

**DOI:** 10.3389/fmed.2022.933884

**Published:** 2022-08-24

**Authors:** E. T. A. M. van Delft, Maha Jamal, Hannah den Braanker, T. M. Kuijper, J. M. W. Hazes, Deirisa Lopes Barreto, A. E. A. M. Weel-Koenders

**Affiliations:** ^1^Department of Rheumatology, Maasstad Hospital, Rotterdam, Netherlands; ^2^Department of Rheumatology, Erasmus Medical Centre, Rotterdam, Netherlands; ^3^Health Technology Assessment, Erasmus University, Rotterdam, Netherlands

**Keywords:** rheumatoid arthritis, referral, outpatient, musculoskeletal complaints, incidence

## Abstract

**Objectives:**

To classify patients with rheumatoid arthritis (RA) in an earlier stage of the disease, the ACR/EULAR classification criteria were updated in 2010. These criteria might have led to an increased incidence of RA in the rheumatology clinic. Since a higher incidence increases the socio-economic burden of RA, it is worthwhile to evaluate whether there is a time effect.

**Materials and methods:**

A systematic review was conducted using Embase, Medline Ovid, Cochrane Central, and Web of Science from database inception to February 2021. Included were only articles that addressed incidence rates of rheumatoid arthritis from rheumatology outpatient clinics.

**Results:**

Of the 6,289 publications only 243 publications on RA were found eligible for full-text review. Nine studies were included reporting incidence. The pooled incidence for RA was 11% (95% CI 6–16%) per year. Over time the incidence increased after the introduction of the 2010 ACR/EULAR classification criteria. Overall there was a high intragroup heterogeneity (*I*^2^ = 97.93%, *p* < 0.001), caused by geographical area, study design and differences in case definitions.

**Conclusion:**

Although the incidence seems to increase after the introduction of the 2010 ACR/EULAR criteria, no conclusions can be drawn on this time effect due to heterogeneity.

## Introduction

About 5% of the population suffers from chronic inflammatory arthritis (IA) (1) of which rheumatoid arthritis (RA) is the most common form (2). The main consequences of RA are painful, swollen, and stiff joints, leading to disability (3). Rheumatoid arthritis has a major impact on socio-economic costs (4), which constitutes a substantial public health issue (5). In the Netherlands the direct healthcare costs for RA are around 0.74% of the entire expenditure on healthcare (6). Next to that, RA also has a major impact on indirect costs, generally resulting from lost productivity (4).

The hallmark in RA treatment is to treat in an early stage with intensive regimens to prevent disability on the longer term (7, 8). Early treatment requires early diagnosis, hence early referral. To facilitate early treatment, updated classification criteria for RA were published in 2010 by a task force of experts from both the European League Against Rheumatism (EULAR) and the American College of Rheumatology (ACR) (9). Compared to older criteria sets for RA these criteria from 2010 cover a broader spectrum of early disease features (10). Compared with the 1987 classification criteria for RA, the 2010 criteria have higher sensitivity but lower specificity (10).

Although classification criteria are developed for use in research and not for the purpose of diagnosing, they are widely used as aids for diagnosing RA. Furthermore the 2010 ACR/EULAR criteria are used commonly in teaching hospitals for trainees (9, 11). Since patients with early arthritis are a very heterogeneous group, the low specificity of the new criteria might cause misclassification when used for diagnosing. Next to that, the criteria also aimed at changing the way professionals look at RA. Therefore, the 2010 ACR/EULAR criteria might have led to an increased reported incidence of RA (12).

Since there is a risk of misclassification due to the use of the 2010 ACR/EULAR criteria, it is of great importance to assess the incidence proportions over time. By conducting a systematic review we aimed to acquire time trends in incidence proportions before and after the introduction of the updated 2010 ACR/EULAR classification criteria within the rheumatology outpatient clinics.

## Materials and methods

This systematic review was conducted in accordance with the Preferred Reporting Items for Systematic Reviews and Meta-Analysis (PRISMA) guidelines (13). The research question was whether a time trend could be seen in incidence proportions of RA in rheumatology clinics after introduction of the 2010 ACR/EULAR classification criteria.

### Literature search

The search strategy was developed in collaboration with an experienced medical librarian of the Erasmus Medical Center, Rotterdam, Netherlands. The digital databases of Embase, Medline, Cochrane, and Web of science were searched to identify relevant studies published from database inception to February 2021. Keywords, indicated as MeSH terms, included terms and synonyms for inflammatory arthritis, prevalence, incidence, and a setting of specialized outpatient secondary or tertiary healthcare. A broad search strategy was established since terms like arthritis, prevalence and incidence are not always used or interpreted uniformly. Therefore the search strategy covered the entire spectrum of inflammatory arthritis to ensure that no articles on RA are missed. The complete search strategy is available in Supplementary file 1.

### Selection criteria

Studies were eligible if they: (i) were written in English language; (ii) included patients aged 18°years or older; (iii) reported the incidence of RA in patients referred to rheumatology outpatient clinics. Studies were excluded if they did not contain original data or had only been published in the form of conference abstracts. There was no restrictive criterion on study design. In case any deviations from the protocol were present, these were clearly reported.

### Data extraction

Inclusion of studies was executed in two stages. First, titles and abstracts were screened for eligibility according to the selection criteria described above. Second, the full text of all articles that had passed the first screening was retrieved to further check the same eligibility criteria. Two reviewers [ED and MJ] screened all titles and abstracts independently and in case of disagreement a third reviewer [HB] was consulted. Following the two-stage inclusion process, ED assessed the full text of half of selected articles and MJ and HB each assessed a quarter of these articles for eligibility. Data were extracted by two investigators [ED and MJ] according to a predefined data form. The following information was extracted: country, setting (secondary care and tertiary care), study design (retrospective and prospective follow-up), number of referred patients participating, mean age, percentage of men, case definition of RA, and number of cases with an RA diagnosis. For any missing information, the authors of the concerning article were contacted to ask for clarification. All data was discussed among the reviewers and disagreements were resolved by consensus after discussion.

### Assessment of methodological quality

All included papers were assessed for methods of data collection by a quality assessment tool for prevalence studies (14). The tool was adjusted for our situation, following the example of Karreman et al. (15). The final list comprised six yes/no questions. Response options for individual items were either low or high risk of bias. If there was insufficient information in the article to permit a judgment for a particular item, then the item was deemed to be at high risk of bias (13). The full quality assessment tool with instructions on how the tool was applied can be found in Supplementary file 2. Agreement between the two raters was assessed using the Kappa statistic. A benefit of using the Kappa statistic is that it takes agreement by chance into account. Kappa values range from −1 to 1, where scores of −1 to 0 indicate poor agreement, 0.01 to 0.20 slight agreements, 0.21 to 0.40 fair agreement, 0.41 to 0.60 moderate agreement, 0.61 to 0.80 substantial agreement, and 0.81 to 0.99 almost perfect agreement (16).

### Analysis

To estimate a time trend incidence, studies were divided into studies before 2010 and 2010, the year in which the ACR/EULAR classification criteria were introduced. Heterogeneity (*I*^2^) was used to address the inconsistency across studies. *I*^2^ describes the proportion of total variation in study estimates that is due to heterogeneity (17). Recommendations were drawn up based on the Grading of Recommendations Assessment, Development and Evaluation (GRADE) approach (18).

## Results

### Search results

**FIGURE 1:**
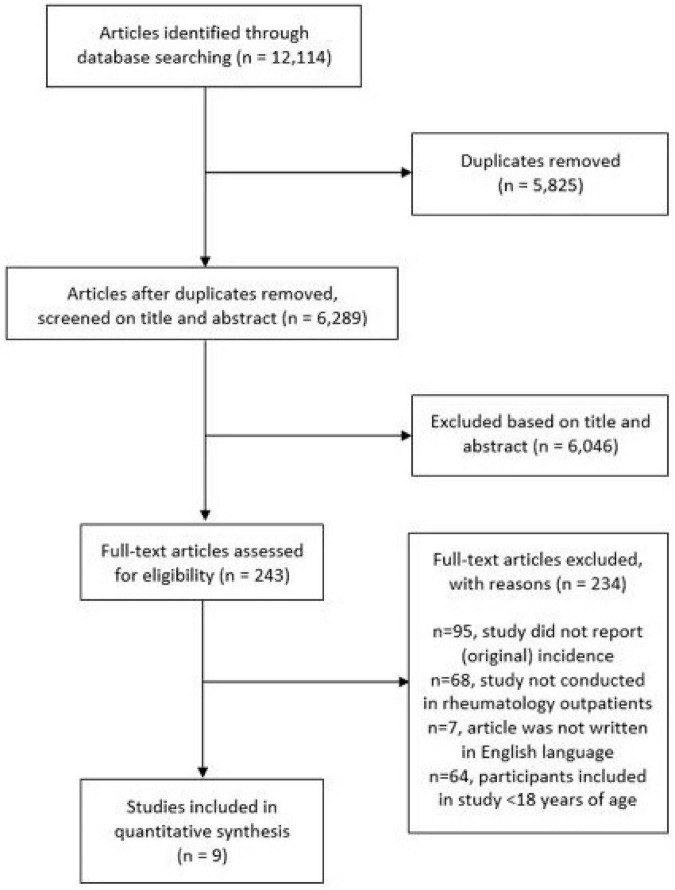
Flow diagram of study selection.

The electronic database search resulted in 12,114 publications (Figure 1). After removal of duplicates and exclusion based on abstract and title, a number of 243 publications were found eligible for full-text review. The majority of studies (*n* = 234) were excluded because the incidence of RA was not reported, data was not originating from an outpatient rheumatology clinic or due to age or language restrictions. In total nine publications were included for analysis. The characteristics of the included studies are shown in Table 1. The reporting on demographic data was incomplete in some of the studies, as well as the reporting on case definition.

**TABLE 1:** Study characteristics of included studies.

Article	Year of publication	Country	Study design	Setting	Patients referred *n*	Mean age [years]	% men	Case definition	Rheumatoid arthritis *n* [%]
Anaya, et al. (19)	2001	Colombia	RS	TC	321	NA	NA	CR (ACR 1987)	16 [4.98]
Benucci, et al. (20)	2008	Italy	PS	TC	920	NA	NA	CR (ACR 1987)	32 [3.48]
Bitik, et al. (21)	2015	Turkey	RS	TC	65	56	26	RD	8 [12.30]
Caines, et al. (22)	2012	Canada	RS	TC	1,101	NA	NA	RD	121 [10.99]
Fonseca, et al. (23)	2018	Portugal	PS	SC	78	47	16.8	RD	3 [3.85]
Holden, et al. (24)	1982	United Kingdom	PS	SC	814	58.9	NA	NA	65 [8.00]
Rais, et al. (25)	2014	Pakistan	RS	SC	2,300	40.3 F 43.7 M	NA	NA	500 [21.70]
Shamim, et al. (26)	2015	Pakistan	PS	TC	316	47.97	26.3	CR (2010 ACR/EULAR)	85 [26.90]
Suarez-Almazor, et al. (27)	1998	Canada	RD	TC	711	49	39	RD	45 [6.00]

NA, not available; RS, retrospective; PS, prospective; TC, tertiary care; SC, secondary care; F, female; M, male; CR, criteria; RD, rheumatologist diagnosis.

### Incidence of rheumatoid arthritis

The incidence of RA in adult patients referred to a rheumatology outpatient clinic was described in nine articles (19–27). The pooled incidence of RA was estimated to be 11% (95% CI 6–16%) per year. A high intragroup heterogeneity was observed between studies (*I*^2^ = 97.93%, *p* < 0.001).

**FIGURE 2:**
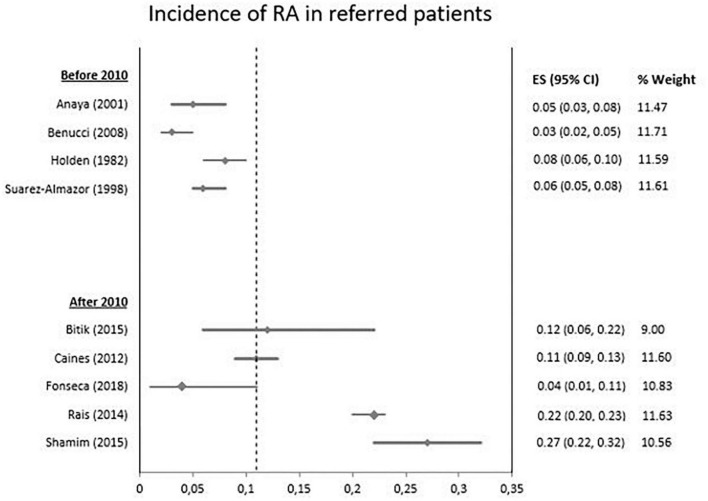
Incidence of rheumatoid arthritis in patients referred to a rheumatologist based on year on study.

Figure 2 takes into account all nine articles and shows a difference in time trend incidence before and after 2010. Four studies reported on the incidence before and five studies after the introduction of the 2010 ACR/EULAR classification criteria.

To determine whether the high pooled incidence after 2010 was related to the differences in geographical area and access to specialized medical care we performed an additional analysis excluding the two Asian studies published after 2010. Then, there was still an increasing time trend in incidence of RA after 2010.

Case definition in the included articles showed great variety both before and after 2010. Whereas before 2010 in 50% of the articles criteria were used to establish RA and in 25% rheumatologist diagnosis was used as a golden standard. After 2010 only 20% of articles used criteria to establish RA and 60% used rheumatologist diagnosis. Variability in the participants and types of case definition is causing clinical heterogeneity.

### Methodological quality of included studies

**FIGURE 3:**
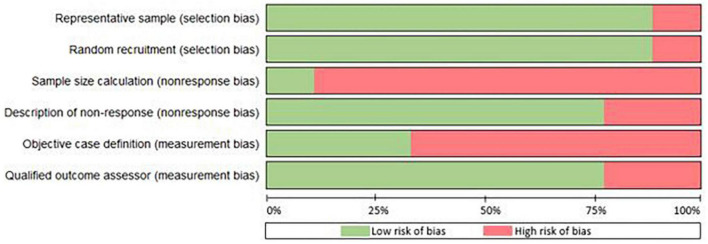
Risk of bias as percentages across the nine included studied in this review.

The Kappa statistic for the overall interrater agreement was 0.81, indicating a very high level of agreement between the two raters. Most of the studies had a sample representative of the target population (89%) and recruited their patients randomly from an appropriate source (89%) (Figure 3). Hence, for one study the sample was not representative and the recruitment was not random, making it subjective to selection bias (21). Only one study reported sample size calculation, although seven out of nine studies did conduct data-analysis with sufficient coverage of the identified sample. With regard to measurement bias, in 33% objective standard criteria were used and in 78% of the studies the outcome assessor was qualified to define cases of RA reliably. The variability in study design and quality is causing methodological heterogeneity. A complete overview of the assessment of methodological quality is found in Supplementary file 3.

## Discussion

In this systematic review we provide insight into the time trend in incidence of RA with respect to the introduction of the 2010 ACR/EULAR classification criteria. An increase in the number of referred patients diagnosed with RA after the introduction of the 2010 ACR/EULAR classification criteria is found.

Whether this increase in incidence is due to an increase of overall disease expression is hard to say. Studies on prevalence before 2010, however, have in fact shown that the prevalence of RA on a population and global level remained stable over the past decades up to 2010 (28–30). Unfortunately not many studies have been performed on the incidence or prevalence of RA after 2010 to compare our findings with.

Most likely, the increase in incidence is related to an increased awareness and recognition of RA since rheumatologists and primary care practitioners have better knowledge and diagnostics to detect the disease. The increased use of the 2010 ACR/EULAR criteria in trainee programs might have by implication swayed more primary care physicians to question a diagnosis of RA and lead to more rheumatology referrals (31). This provides rheumatologists with the opportunity to classify RA more frequently. On top of that, more sensitive diagnostic methods and the availability of the 2010 ACR/EULAR classification criteria might have increased the number of RA patients (32).

While in practice the classification criteria are used as aids in diagnosing, they were not developed for the purpose of being used as diagnostic criteria or as a referral tool for primary care (6). Classification criteria are primarily intended to create well-defined, relatively homogenous cohorts for clinical research. On the contrary, diagnostic criteria are generally broad and must reflect the heterogeneity of a disease (33). This makes classification criteria inappropriate for use as aids in diagnosing in daily clinical practice (34) and thus neither as means to determine the incidence of RA. In this review studies are included in which both rheumatologist diagnosis and classification criteria are used to establish RA. Luckily there appears to be a shift toward diagnosing merely based on rheumatologist diagnosis as a golden standard, opposed to using inappropriate classification criteria for diagnosing.

We show that the reported incidence is influenced by a large heterogeneity. However, after excluding the two Asian studies by Rais et al. and Shamim et al. that were conducted in Pakistan after 2010, the incidence is still higher when we compare the incidence before and after 2010. The high incidence in the Shamim study (26) might have been influenced by the use of the 2010 ACR/EULAR criteria. Another influencing factor might have been the difference in access to specialized medical care in Pakistan compared to other countries included in this review. The specialist referral in Pakistan is patient-driven (25, 35), most people access secondary or tertiary care hospitals directly. Whereas in other countries there is a strict referral system in which patients need referral through primary care before visiting a rheumatologist.

The quality assessment of the included studies shows that there is large variety in methodological quality of studies. Most studies score positive on four out of six items of the quality assessment tool; however there are also studies that score less than three positive items. Additionally, the reporting on demographic data or case definition is incomplete in some of the studies. Unfortunately, not all continents are represented in this study and some demographic data are absent which does not allow for inferences on general population characteristics. There might be some indication bias due to the fact that referral systems differ across the globe. In this review only articles are included in which diagnoses are made by a rheumatologist, while in some countries RA is already diagnosed in primary care. These methodological issues might have affected the results of studies in the comparison between the occurrence of the disease among different countries or when analyzing the time trends (36). The results of this review are therefore only generalizable to countries with a similar referral system in which patients are referred from primary care toward a rheumatologist.

Several strengths of the current review should be taken into account. This systematic review is conducted in accordance with the Preferred Reporting Items for Systematic Reviews and Meta-Analysis (PRISMA) guidelines (13). An extensive search strategy was set up in collaboration with an experienced librarian in order to identify as many relevant studies as possible. The decision to include terms and synonyms for both prevalence and incidence has enhanced our results, since in literature prevalence and incidence are often used interchangeably. A risk of bias assessment is also included to give an indication of the methodological quality of the included studies. The risk of bias tool that is used was initially developed for prevalence studies only. However, since detailed criteria and examples were given for each item of this tool, we were able to select items that were applicable to incidence studies. Having evaluated the quality of evidence precisely helps strengthen recommendations (37). The entire selection of studies, data extraction and assessment of methodological quality were conducted by two independent reviewers and every paper was discussed until full consensus was reached. Nevertheless, it is important to note that updating a systematic review periodically is recommended (38).

For future research into incidence of inflammatory rheumatic diseases, we do have some recommendations. To overcome methodological issues, it is of great importance to use an objective case definition to overcome measurement bias. Next to that, the case definition should be clearly reported in the article, as well as crucial data like demographic parameters of the study population. As a final recommendation, we would encourage researchers to clearly look at whether the study investigates the prevalence or the incidence of a certain condition, since both terms are often used interchangeably. Within the era we live in at the moment, with digital revolution happening at high speed, this adequate data registration is not only important for research purposes, but overall to ensure real life hospital data transparency.

A clinical implication following from this review might be to conclude that the workload for rheumatologists increases equivalently with the increasing incidence of RA. For society this would mean increasing healthcare costs. However, as mentioned the 2010 ACR/EULAR criteria may sway primary care physicians to consciously question a diagnosis of RA and be more cautious on whom to refer (31). Next to that, numerous initiatives are being conducted at the moment with the aim of improving appropriateness of referrals toward the rheumatologist (39). It is our experience that around 70% of all patients referred to an outpatient rheumatology clinic is not diagnosed with an inflammatory rheumatic disease. While with a smaller number of inappropriate referrals, rheumatologists can spend more of their time on patients with an inflammatory rheumatic disease. This may outbalance the increasing number of RA patients and allows starting treatment in an early stage of the disease to overcome progression. Next to that the increase of appropriateness of referrals may also have socio-economic advantages.

In conclusion, an increased incidence of RA in the outpatient rheumatology clinic is seen after 2010 compared to earlier studies. However, due to the large heterogeneity between studies, this increase cannot be fully attributed to the introduction of the 2010 ACR/EULAR classification criteria. Although it is stated that these criteria lead to better and earlier recognition of RA, further research with coherent use of the 2010 ACR/EULAR criteria is needed to establish the diagnostic effects in daily clinical practice worldwide.

## Data availability statement

The original contributions presented in this study are included in the article/Supplementary material, further inquiries can be directed to the corresponding author.

## Author contributions

ED, JH, DL, and AW-K contributed to conception and design of the study. ED, MJ, and HB performed data collection. ED and TK performed the statistical analysis. ED wrote the first draft of the manuscript. All authors contributed to manuscript revision, read, and approved the submitted version.
