# Supplementary material for: A systematic review on time trend incidence of rheumatoid arthritis in outpatient rheumatology clinics
Source: Front Med (Lausanne). 2022 Aug 24;9:933884. doi: 10.3389/fmed.2022.933884 (PMC9448917; doi:10.3389/fmed.2022.933884)
Supplement: Supplementary file 1 [file Data_Sheet_1.docx]

Supplementary Material

1. **Supplementary Data S1; Search strategy (2021-02-09)**

**Embase.com**

('rheumatoid arthritis'/de OR 'spondylarthritis'/de OR 'psoriatic arthritis'/de OR 'ankylosing spondylitis'/de OR 'rheumatic disease'/de OR 'rheumatology'/de OR 'rheumatologist'/de OR (alibert-bazin OR beauvais-disease* OR ((arthrit* OR osteoarthrit*) NEAR/3 (deforman* OR inflammat* OR rheumat* OR vertebr* OR spina* OR psoria*)) OR rheumarthrit* OR spondylarthrit* OR spondyloarthrit* OR (ankylos* NEAR/3 spondylit*) OR (Rheumatic NEAR/3 (disease* OR disorder*)) OR rheumatolog* OR reumatolog* OR ((rheuma* OR arthralgi*) NEAR/3 specialist*)):ab,ti) AND ('incidence'/de OR 'prevalence'/de OR 'statistics'/de OR (incidence* OR prevalence* OR statistic* OR ((spectrum OR pattern* OR occurenc* OR frequenc* OR percent* OR proportion*) NEAR/6 (disease* OR inflammat*)) OR ((spectrum OR pattern* OR distribution) NEAR/3 patient*) ):ab,ti) AND ('outpatient'/exp OR 'outpatient department'/exp OR 'secondary health care'/exp OR 'tertiary health care'/exp OR 'ambulatory care'/de OR 'patient referral'/de OR (outpatient* OR out-patient* OR ((secondar* OR tertiar* OR specialist OR public) NEAR/3 (care* OR healthcare OR hospital OR center* OR centre*)) OR ((rheumat* OR reumat* OR Arthrit*) NEAR/3 (clinic OR department* OR centre* OR center* OR institut* OR unit OR units)) OR ((rheumat* OR reumat* OR Arthrit*) NEXT/1 practice*) OR ambulator* OR referr*):ab,ti) NOT ([Conference Abstract]/lim OR [Letter]/lim OR [Note]/lim OR [Editorial]/lim) AND [english]/lim

**Medline Ovid**

(Arthritis, Rheumatoid/ OR Spondylarthritis/ OR Arthritis, Psoriatic/ OR Spondylitis, Ankylosing/ OR Rheumatic Diseases/ OR Rheumatology/ OR Rheumatologists/ OR (alibert-bazin OR beauvais-disease* OR ((arthrit* OR osteoarthrit*) ADJ3 (deforman* OR inflammat* OR rheumat* OR vertebr* OR spina* OR psoria*)) OR rheumarthrit* OR spondylarthrit* OR spondyloarthrit* OR (ankylos* ADJ3 spondylit*) OR (Rheumatic ADJ3 (disease* OR disorder*)) OR rheumatolog* OR reumatolog* OR ((rheuma* OR arthralgi*) ADJ3 specialist*)).ab,ti.) AND (incidence/ OR prevalence/ OR (incidence* OR prevalence* OR statistic* OR ((spectrum OR pattern* OR occurenc* OR frequenc* OR percent* OR proportion*) ADJ6 (disease* OR inflammat*)) OR ((spectrum OR pattern* OR distribution) ADJ3 patient*) ).ab,ti.) AND (Outpatients/ OR Outpatient Clinics, Hospital/ OR Secondary Care/ OR Tertiary Healthcare/ OR Ambulatory Care/ OR " Referral and Consultation "/ OR (outpatient* OR out-patient* OR ((secondar* OR tertiar* OR specialist OR public) ADJ3 (care* OR healthcare OR hospital OR center* OR centre*)) OR ((rheumat* OR reumat* OR Arthrit*) ADJ3 (clinic OR department* OR centre* OR center* OR institut* OR unit OR units)) OR ((rheumat* OR reumat* OR Arthrit*) ADJ practice*) OR ambulator* OR referr*).ab,ti.) NOT (letter* OR news OR comment* OR editorial* OR congres* OR abstract* OR book* OR chapter* OR dissertation abstract*).pt. AND english.la.

**Cochrane CENTRAL**

((alibert-bazin OR beauvais-disease* OR ((arthrit* OR osteoarthrit*) NEAR/3 (deforman* OR inflammat* OR rheumat* OR vertebr* OR spina* OR psoria*)) OR rheumarthrit* OR spondylarthrit* OR spondyloarthrit* OR (ankylos* NEAR/3 spondylit*) OR (Rheumatic NEAR/3 (disease* OR disorder*)) OR rheumatolog* OR reumatolog* OR ((rheuma* OR arthralgi*) NEAR/3 specialist*)):ab,ti) AND ((incidence* OR prevalence* OR statistic* OR ((spectrum OR pattern* OR occurenc* OR frequenc* OR percent* OR proportion*) NEAR/6 (disease* OR inflammat*)) OR ((spectrum OR pattern* OR distribution) NEAR/3 patient*) ):ab,ti) AND ((outpatient* OR out-patient* OR ((secondar* OR tertiar* OR specialist OR public) NEAR/3 (care* OR healthcare OR hospital OR center* OR centre*)) OR ((rheumat* OR reumat* OR Arthrit*) NEAR/3 (clinic OR department* OR centre* OR center* OR institut* OR unit OR units)) OR ((rheumat* OR reumat* OR Arthrit*) NEXT/1 practice*) OR ambulator* OR referr*):ab,ti)

**Web of science**

TS=(((alibert-bazin OR beauvais-disease* OR ((arthrit* OR osteoarthrit*) NEAR/2 (deforman* OR inflammat* OR rheumat* OR vertebr* OR spina* OR psoria*)) OR rheumarthrit* OR spondylarthrit* OR spondyloarthrit* OR (ankylos* NEAR/2 spondylit*) OR (Rheumatic NEAR/2 (disease* OR disorder*)) OR rheumatolog* OR reumatolog* OR ((rheuma* OR arthralgi*) NEAR/2 specialist*))) AND ((incidence* OR prevalence* OR statistic* OR ((spectrum OR pattern* OR occurenc* OR frequenc* OR percent* OR proportion*) NEAR/5 (disease* OR inflammat*)) OR ((spectrum OR pattern* OR distribution) NEAR/2 patient*) )) AND ((outpatient* OR out-patient* OR ((secondar* OR tertiar* OR specialist OR public) NEAR/2 (care* OR healthcare OR hospital OR center* OR centre*)) OR ((rheumat* OR reumat* OR Arthrit*) NEAR/2 (clinic OR department* OR centre* OR center* OR institut* OR unit OR units)) OR ((rheumat* OR reumat* OR Arthrit*) NEAR/1 practice*) OR ambulator* OR referr*))) AND DT=(article) AND LA=(english)

**Google scholar**

rheumatologist|reumatologist|"rheumatology|arthralgia specialist |specialists" incidence|prevalence|statistics|spectrum|pattern|frequency inflammation|inflammatory outpatient|"secondary|tertiary|specialist care|healthcare|hospital|center|centre"

1. **Supplementary Table S2; Risk of bias assessment instructions**

|  |  | YES | NO |
| --- | --- | --- | --- |
| 1. | Was the sample representative of the target population? | The sample was representative of the target population. Selected patients are representative of a population of patients suspected of RA. No pre-selection took place in selecting the patients based on for example work.  The center from which the RA patients were recruited should me mentioned | Sample was not representative. |
| 2. | Were study participants recruited in an appropriate way? | Patients were recruited from an appropriate source and were “randomly” invited for the study (all patients OR consecutive patients OR random patients) | Patients were not recruited from an appropriate source and no random selection was used to recruit patients |
| 3. | Was the sample size adequate / Was sample size calculation performed? | Sample size calculation was performed and it was reported if this target was reached | No sample size calculation |
| 4. | Was the data analysis conducted with sufficient coverage of the identified sample? | Non-response was described **AND** a comparison between the responders and non-responders was performed.  If retrospective design, answer is yes | No information about response percentages was given or no comparison between responders and non-responders was made. |
| 5. | Were objective, standard criteria used for the measurement of the condition? | Criteria were used for the diagnosis of RA (for example ACR or EULAR criteria)  OR  A detailed description of how a case of RA was defined is included in the manuscript.  OR  In case of use of ICD codes, a validation/check was performed | No criteria were used and no description of how a case was defined is included in the manuscript. |
| 6. | Was the condition measured reliably? | Outcome assessor was qualified to use the case definition criteria (for example; medical specialist, trained research nurse) | Outcome assessor was not qualified to use the case definition criteria or it was not mentioned who defined a case. |

1. **Supplementary Table S3; Overview of assessment of methodological quality**

|  | Anaya, 2001 | Benucci, 2008 | Bitik, 2015 | Caines, 2012 | Fonseca, 2018 | Holden, 1982 | Rais, 2014 | Shamim, 2015 | Suarez-Almazor, 1998 |
| --- | --- | --- | --- | --- | --- | --- | --- | --- | --- |
| Representative sample  (selection bias) | 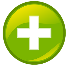 |  | 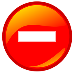 |  |  |  |  |  | 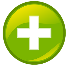 |
| Random recruitment (selection bias) |  |  | 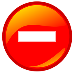 |  |  |  | 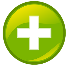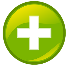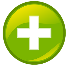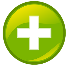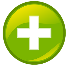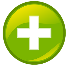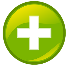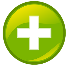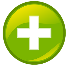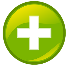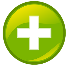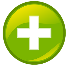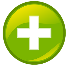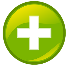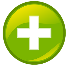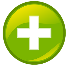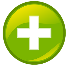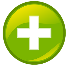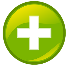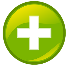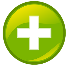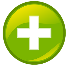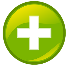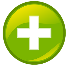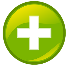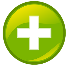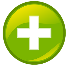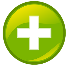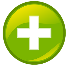 |  | 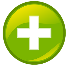 |
| Sample size calculation (nonresponse bias) | 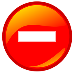 | 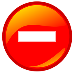 | 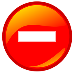 | 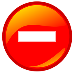 |  | 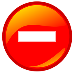 | 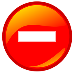 | 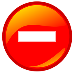 | 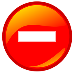 |
| Description of non-response (nonresponse bias) |  | 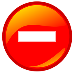 |  |  |  | 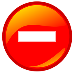 |  |  | 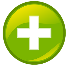 |
| Objective case definition (measurement bias) |  |  | 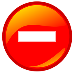 | 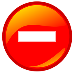 | 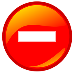 | 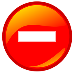 | 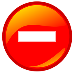 |  | 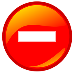 |
| Qualified outcome assessor (measurement bias) |  |  |  |  |  | 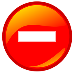 | 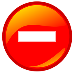 |  | 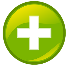 |


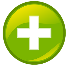
 Low risk of bias
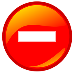
 High risk of bias
